# Challenges in translating vascular tissue engineering to the pediatric clinic

**DOI:** 10.1186/2045-824X-3-23

**Published:** 2011-10-14

**Authors:** Daniel R Duncan, Christopher K Breuer

**Affiliations:** 1 Interdepartmental Program in Vascular Biology and Therapeutics, Yale University School of Medicine, 10 Amistad Street, New Haven, CT 06520, USA

**Keywords:** Tissue-engineered vascular grafts, congenital heart disease, translational research

## Abstract

The development of tissue-engineered vascular grafts for use in cardiovascular surgery holds great promise for improving outcomes in pediatric patients with complex congenital cardiac anomalies. Currently used synthetic grafts have a number of shortcomings in this setting but a tissue engineering approach has emerged in the past decade as a way to address these limitations. The first clinical trial of this technology showed that it is safe and effective but the primary mode of graft failure is stenosis. A variety of murine and large animal models have been developed to study and improve tissue engineering approaches with the hope of translating this technology into routine clinical use, but challenges remain. The purpose of this report is to address the clinical problem and review recent advances in vascular tissue engineering for pediatric applications. A deeper understanding of the mechanisms of neovessel formation and stenosis will enable rational design of improved tissue-engineered vascular grafts.

## The Tissue Engineering Approach

Tissue engineering offers a strategy for constructing autologous grafts and thereby increasing the pool of potential autografts for use as vascular conduits [1]. Using the classical tissue engineering paradigm, autologous cells can be seeded onto a biodegradable tubular scaffold, which provides sites for cell attachment and space for neotissue formation [2]. As the neotissue forms, the scaffold degrades creating a purely biological graft. The resulting neotissue can thus function as a vascular graft in cardiothoracic operations [3]. Extensive large animal studies have demonstrated the feasibility of using tissue engineering methodology to construct conduits for use as large grafts [3-6].

Research groups have used a variety of different approaches to develop tissue-engineered vascular grafts (TEVG). Several methods are now in use in the lab and at various stages of clinical development. These include in vivo engineering of blood vessels, using explanted native vessels as a living scaffold for tissue engineering, a variety of biodegradable polymeric scaffolds onto which cell types can be seeded, and scaffold-free approaches [7-9]. The ideal tissue-engineered vascular conduit is not yet in use and when it comes to optimizing the translation of this emerging technology, all elements of the process of TEVG development need to be considered including scaffold materials, cells for seeding grafts, and seeding techniques.

### Scaffold Materials

Scaffold materials must not only be biodegradable and non-immunogenic, but also must provide space for cell attachment while allowing for appropriate structural integrity until neotissue can form. Standard approaches involve the use of polymers of polyglycolic acid (PGA), polylactic acid (PLA), and poly e-caprolactone (PCL) in varying concentrations to meet the compliance specifications of the vascular system into which the graft is being introduced [10,11]. Electrospinning is a newer approach for creating vascular graft scaffolds that can be made with finely tuned biomechanical specifications [12]. Other groups have pioneered the use of decellularized biologic materials including human and porcine vessels [13]. Additional novel approaches involve the use of human umbilical vein as a living scaffold and grafts made using sheets of a patient's own fibroblasts [7-9].

### Cells for Seeding

Many cell types have been considered as possibilities for seeding vascular grafts [14,15]. Some groups have investigated the use of endothelial cells and smooth muscle cells for seeding but these approaches require long incubation times, presenting additional risk of contamination along with delaying the implantation. Recent investigation has focused on shortening the time required for this approach, including the use of novel flow chambers and other bioreactors [15].

Bone marrow mononuclear cells have been found to be a useful cell source as they are readily available from patients by means of bone marrow aspiration. There are several different approaches for purifying mononuclear cells from the bone marrow. The traditional approach has involved Ficoll centrifugal separation based on cell mass, but this takes several hours. A newer approach involves using a specially designed filter to separate out cells of a particular size [16]. Alternative methods need to optimize speed and specificity for the cells of interest, while maintaining sterility and cell viability.

Alternative cell sources that might provide additional benefits include embryonic stem (ES) cells or induced pluripotent stem (iPS) cells, the latter offering a new autologous approach to developing pluripotent cells [17-19]. All pluripotent cells present the risk of teratoma formation and so more investigation is needed into the use of these cell types for the seeding of TEVGs. It is yet to be seen whether an optimal approach would involve seeding with undifferentiated ES or iPS cells or rather using these cells derived from a patient to make a differentiated cell line of smooth muscle and/or endothelial cells before the seeding of vascular grafts [20].

### Seeding Techniques

The traditional approach to placing cells on a scaffold for TEVG creation is static cell seeding, in which the patient's cells are pipetted directly onto a graft before being given several hours to attach. There are a number of recognized shortcomings of the static seeding method, including lower efficiency and inter-operator variability. A number of alternatives have been proposed, including dynamic, magnetic, vacuum, electrostatic, and centrifugal seeding [21]. The leading option at this point seems to be vacuum seeding in a specially designed chamber, which is both more standardized and more effective in that it allows for rapid, operator-independent, and self-contained cell seeding [22].

## Clinical Background

It will be important to have a deeper understanding of the mechanisms of neotissue formation and stenosis for an upcoming FDA approved clinical trial that is to be initiated at Yale School of Medicine to investigate the use of TEVGs in pediatric patients [23,24]. The development of tissue-engineered vascular grafts for use in cardiovascular surgery holds particular promise for improving outcomes in pediatric patients with complex congenital cardiac anomalies.

Despite major advances in medical and surgical treatment, congenital heart disease (CHD) remains the leading cause of death due to congenital anomalies in the newborn period [25]. Single ventricle anomalies make up one of the largest groups of cardiac anomalies resulting in life-threatening diseases. These include diseases such as tricuspid atresia, pulmonary atresia, and hypoplastic left heart syndrome, in which only one ventricle is of adequate functional size. These anomalies result in mixing of the deoxygenated pulmonary circulation and the oxygenated systemic circulation, causing chronic hypoxia and cyanosis. This mixed circulation can cause volume overload that can lead to heart failure. Untreated single ventricle anomalies are associated with up to 70% mortality during the first year of life [26]. The treatment of choice for this CHD is surgical reconstruction, the goal of which is to separate the pulmonary circulation from the systemic circulation [27,28]. This is accomplished through a series of staged procedures referred to as the modified Fontan operation with extra cardiac total cavopulmonary connection (EC TCPC). This operation has considerably improved long-term survival but is considered only a palliative procedure with significant morbidity and mortality [27,28].

An important cause of complications in EC TCPC is the conduit used to connect the inferior vena cava (IVC) to the pulmonary artery [29]. Much of the late morbidity is attributed to problems with conduit use [30] and while as many as 10,000 children undergo such reconstructive cardiothoracic operations each year, it is widely accepted that the ideal conduit has not yet been developed [31-33]. Data describing long-term graft failure rates for conduits used for EC TCPC is limited but long-term data for similar congenital heart conduit operations suggest outcomes are poor [34]. Late problems include conduit degeneration with progressive obstruction and susceptibility to infection. Synthetic conduits are also a significant cause of thromboembolic complication due to the area of synthetic material in contact with blood causing activation of the coagulation cascade [35]. Synthetic conduits lack growth potential, necessitating re-operation when a pediatric patient outgrows the graft. Re-operation is associated with significant morbidity and early post-operative mortality rates as high as 5% [34]. Long-term graft failure rates have been reported at 70-100% at 10-15 years [36,37]. The best results have been obtained when autologous tissue was used for the conduit with long-term patency rates of over 80% [38]. Autografts, conduits created from an individual's own tissue, have better long-term effectiveness than any synthetic or biological conduit currently available but these are limited in supply, suggesting the need for an alternate approach [34,37-39].

## Clinical Trial

**Figure 1:**
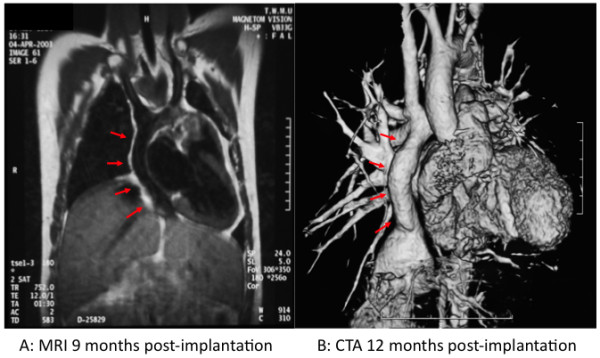
**Growth potential of TEVG in clinical trial**. A. Magnetic resonance image (MRI) 9 months following implantation of EC TCPC graft. B. 3-D computed tomography angiogram (CTA) of graft one year after implantation. Red arrows indicate location of tissue-engineered vascular graft. (Adapted with permission from Shinoka (2008) [23]).

Based on the success of animal studies, Shinoka performed a pilot clinical study in Japan in 2001 to evaluate the feasibility and safety of using TEVG as conduits for EC TCPC in patients with single ventricle cardiac anomalies [40-42]. Twenty-five TEVG seeded with autologous bone marrow mononuclear cells (BM-MNC) were implanted with follow-up out through seven years [4,43]. At the most recent follow-up, the tissue-engineered vascular grafts were shown to function well without evidence of graft failure. No graft had to be replaced and there was no graft related mortality. An additional advantage of this technology is almost eliminating the need for antiplatelet, antigoagulant, and immunosuppressive therapy. All patients had both antiplatelet and anticoagulant medications discontinued by 6 months postoperatively and 40% of patients remained free of any daily medications long term in stark contrast to the lifetime need for anticoagulation with the use of synthetic grafts [40]. Long-term follow-up, however, revealed graft stenosis in 16% of patients (Table 1). Stenosis in these patients was frequently asymptomatic and all were successfully treated with angioplasty and stenting. In addition, serial imaging demonstrated the growth potential of these grafts, an element that is extremely important in the pediatric population (Figure 1). These data support the overall feasibility and safety of using vascular tissue engineering technology in the pediatric clinical setting [40].

**Table 1:** Late term status after TEVG implantation in clinical trial

Patient	Age at Operation (Years)	Patient Status	Graft Status	Graft Patency	Graft Related Complications
1	2	alive	intact	patent	none
2	1	alive	intact	patent	none
3	7	alive	intact	patent	stenosis
4	21	alive	intact	patent	none
5	4	alive	intact	patent	none
6	12	alive	intact	patent	none
7	17	alive	intact	patent	none
8	19	dead	intact	patent	none
9	3	alive	intact	patent	stenosis
10	2	dead	intact	patent	none
11	13	alive	intact	patent	stenosis
12	2	dead	intact	patent	none
13	2	alive	intact	patent	thrombosis
14	2	alive	intact	patent	none
15	2	alive	intact	patent	none
16	2	alive	intact	patent	none
17	24	alive	intact	patent	none
18	1	alive	intact	patent	stenosis
19	11	alive	intact	patent	none
20	2	alive	intact	patent	none
21	3	alive	intact	patent	none
22	4	alive	intact	patent	none
23	4	alive	intact	patent	none
24	13	alive	intact	patent	none
25	2	dead	intact	patent	none

Most recent follow-up at mean of 5.8 years showed no graft-related mortality and no evidence of aneurysm formation, graft rupture, or ectopic calcification. 4 out of 25 patents developed asymptomatic stenosis that was picked up on routine serial imaging and were successfully treated with angioplasty. All implanted TEVG are currently intact and patent. (Adapted from Hibino (2010) with permission from Elsevier [40]).

Complications arising from the use of currently available synthetic vascular grafts are a leading cause of morbidity and mortality after congenital heart surgery [29]. The lack of growth potential of synthetic conduits is problematic. Use of over-sized grafts in an attempt to avoid outgrowing a conduit is widely practiced, but graft over-sizing has an increased risk of complications [44]. Delaying surgery to minimize re-operations can lead to cardiac dysfunction or heart failure due to prolonged exposure to volume overload and chronic hypoxia [35]. The development of a vascular graft with growth potential would eliminate this problem. Review of the data suggest that the safety and efficacy of the use of TEVG in congenital heart surgery is excellent, but mechanisms underlying the process of neovessel formation that lead to TEVG failure have remained incompletely understood. Exploring these processes is essential to create an improved tissue-engineered vascular conduit. Also, as noted at long-term follow-up, it was found that the primary mode of failure for TEVG is stenosis [3-6,40,43]. Identification of the mediators of stenosis in TEVG and determination of the mechanisms underlying neovessel formation would identify targets and potential strategies for preventing stenosis and thereby enable the rational design of improved TEVG.

## Mechanisms of Neotissue Formation

### Neotissue Growth

In order to better study the mechanisms of TEVG formation and stenosis in vivo, mouse models have been developed to recapitulate the results of the human trial. This approach includes a method for constructing sub-1 mm tubular scaffolds similar to the scaffold used in the clinical trial [45]. These scaffolds can be seeded with cells to create TEVG. Use of immunodeficient SCID-beige mice has enabled transplantation of human cells or cells from strains of transgenic mice without the need for immunosuppression. This has proven to be an excellent model for evaluating TEVG [46,47]. In an initial pilot study, TEVG were implanted as infrarenal IVC interposition grafts and observed over a six-month time course to determine the effect of human BM-MNC seeding on neovessel formation. The seeded TEVG functioned well and had better long-term graft patency and less stenosis than the unseeded scaffolds [48]. Quantitative morphometric analysis demonstrated that unseeded TEVG had significantly increased wall thickness and luminal narrowing compared to seeded TEVG. Further analysis revealed that the primary mode of failure was stenosis characterized by graft wall thickening and progressive luminal narrowing, which ultimately led to luminal obliteration and vessel occlusion by inward remodeling. Cell seeding appeared to inhibit inward remodeling and promote outward remodeling in neovessel formation [48].

A series of time course experiments using ovine and canine models demonstrated the stepwise morphologic changes and graft growth that occur when a seeded scaffold is implanted as a vascular interposition graft [4-6,49]. The process begins with a host-derived inflammatory response followed by formation of a monolayer of endothelial cells lining concentric layers of smooth muscle that develop on the luminal surface of the scaffold. As the scaffold degrades, the cells produce an extracellular matrix rich in collagen, elastin, and glycosaminoglycans, resulting in the formation of a neovessel with biomechanical properties similar to native blood vessel complete with intimal, medial, and adventitial layers that histologically resemble native vessel. The vascular neotissue shows evidence of normal growth and development including increase in size proportional to the surrounding native tissue and expression of Ephrin B4, the molecular determinant of veins, when implanted as an IVC graft [49].

### Neotissue Remodeling

Extensive histological and immunohistochemical (IHC) characterization has been performed to show the changes in TEVG over time in a murine model and these have documented the natural history of neovessel formation, the process of developing from a biodegradable tubular scaffold seeded with BM-MNC into a vascular conduit that resembles a native blood vessel. Six-months after implantation, the resulting neotissue possesses an internal monolayer of endothelial cells surrounded by inner smooth muscle layers, and an organized extracellular matrix. Some groups have hypothesized that stem cells within the bone marrow cell population differentiate into the cells of the neotissue [50]. However, characterizing the human BM-MNC population revealed very few endothelial cells, smooth muscle cells and vascular progenitor cells and therefore it seemed that the seeded cells were unlikely to be the ultimate source of the vascular neotissue. This hypothesis is not consistent with classic tissue engineering theory, which views the seeded cells as building blocks of neotissue, but instead supports a regenerative medicine paradigm in which the seeded scaffold is used to augment the body's own reparative mechanisms to "regenerate" missing tissue. To test this hypothesis, species-specific IHC stains were used to determine the fate of the seeded human BM-MNC in the mouse host. Results of these studies revealed that seeded cells were replaced by host cells one to three-weeks after implantation. These findings were confirmed using human specific GAPDH RNA detection via RT-PCR, which validated the presence of human RNA on the TEVG prior to implantation. This was followed by a dramatic decrease such that no human RNA could be found by post-operative day 7 [48].

**Figure 2:**
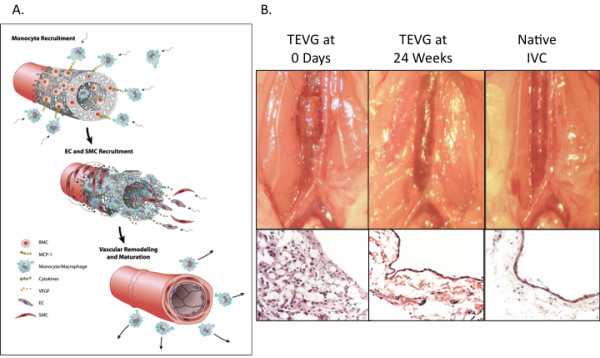
**TEVG remodeling in a mouse model**. A. Inflammation-mediated process of graft remodeling. Seeded BM-MNC attach to the scaffold and release cytokines. MCP-1 recruits host monocytes which infiltrate the scaffold and begin to direct neotissue formation, ultimately resulting in the formation of neovessels composed of a concentric layers of smooth muscle cells recruited from the neighboring native vessel wall embedded in an extracellular matrix with a monolayer of endothelial cells lining the luminal surface. B. TEVG gross and microscopic morphology changes over time and ultimately resembles the native IVC with a smooth muscle cell layer lined by an endothelial cell layer as shown in gross images and hematoxylin and eosin stained section slides. (Adapted with permission from Roh (2010) [48]).

Based on these preliminary studies it has been hypothesized that seeded cells exert their effect via a paracrine mechanism by releasing chemokines that recruit host cells to the scaffold. These host cells are then critical for vascular neotissue formation and promote outward remodeling to maintain graft patency. IHC characterization demonstrated that the TEVG were initially infiltrated by host-derived monocytes and macrophages. Based on quantitative IHC data a correlation was noted between degree of early inflammatory response and graft patency. Specifically, the seeded grafts had significantly more macrophages in the early period compared to unseeded vascular grafts, suggesting that macrophage recruitment may be important in the process of promoting outward remodeling during neovessel formation. IL-1β and MCP-1 were found to be produced in abundant quantity. Studies have been conducted on TEVG seeded with BM-MNC from either MCP-1 knockout mice or wild-type. These TEVG implanted into a SCID-beige vascular interposition graft model revealed that TEVG seeded with MCP-1 knockout BM-MNC developed significantly more wall thickening and luminal narrowing, suggesting that MCP-1 plays a critical role in inducing outward remodeling. Alginate microspheres were created and incorporated into the wall of the scaffold to provide controlled release of MCP-1. A study using this scaffold showed that an MCP-1 eluting scaffold can inhibit stenosis in the absence of BM-MNC seeding. These studies suggest that BM-MNC scaffolds transform into functional vessels by means of an inflammation-mediated process of vascular remodeling (Figure 2) [48].

According to this model, the seeded BM-MNC attach to the scaffold and begin to release MCP-1. Once implanted as an IVC interposition graft, MCP-1 recruits host monocytes, which infiltrate the scaffold and begin to direct or participate in vascular neotissue formation. This remodeling ultimately results in the formation of neovessels composed of a concentric layers of smooth muscle cells recruited from the neighboring native vessel wall embedded in an extracellular matrix with a monolayer of endothelial cells lining the luminal surface [48]. Recent studies have focused on determining the source of neotissue cells. These studies used composite grafts consisting of male vessel segments that were implanted into female mice and wildtype mice given GFP bone marrow transplants. These studies showed that the cells of the neovessel do not derive from the bone marrow or the seeded cells but actually arise as a result of migration from the adjacent vessel segment as an augmented regenerative response [51].

## Conclusions: Improving Clinical Outcomes

The findings of Shinoka's clinical trial in Japan are encouraging but also point to some of the possible issues with the use of vascular grafts in the pediatric population. Translational research groups can now return to animal models in the lab to improve TEVG outcomes [24]. Further investigation will identify critical mediators controlling the formation of stenosis in TEVG. An important goal is to use these discoveries to guide rational design of second-generation TEVG: first, by targeting critical mediators of stenosis, the primary cause of TEVG failure, in order to design grafts with improved long-term patency; and second, by elucidating molecular mechanisms that control vascular neotissue formation in order to create cytokine-eluting TEVG, which would not require cell seeding. The development of a TEVG that does not require cell seeding would improve the off-the-shelf availability of TEVG and dramatically increase its clinical utility.

## Abbreviations

BM-MNC: Bone marrow mononuclear cells; CHD: Congenital Heart Disease; EC TCPC: Extra Cardiac Total Cavopulmonary Connection; IVC: Inferior vena cava; TEVG: Tissue-engineered vascular graft.

## Competing interests

CKB receives research funding from Gunze Ltd, the company that manufactured the scaffolds for the clinical trial. None of the funding for the work done in this manuscript was provided by Gunze Ltd.

## Authors' contributions

DRD and CKB both reviewed the literature and drafted the manuscript. Both authors read and approved the final manuscript.

## Authors' information

DRD is a Howard Hughes Medical Institute Medical Research Training Fellow and CKB is an Associate Professor of Surgery and Pediatrics and the Director of Tissue Engineering at Yale University School of Medicine.
